# Use of complementary and alternative medicine by older adults – a cross-sectional survey

**DOI:** 10.1186/1471-2318-14-38

**Published:** 2014-03-26

**Authors:** Katharina Schnabel, Sylvia Binting, Claudia M Witt, Michael Teut

**Affiliations:** 1 Institute for Social Medicine, Epidemiology and Health Economics, Charité Universitätsmedizin, D-10098 Berlin, Germany; 2 Institute for Complementary and Integrative Medicine, University Hospital Zurich, Zurich, Switzerland; 3 University of Maryland School of Medicine, Center for Integrative Medicine, Baltimore, USA

**Keywords:** Older adults, CAM, Dietary supplements, Nursing home, Residential care, Legal guardian

## Abstract

**Background:**

Very little is known about complementary and alternative medicine (CAM) use by older adults in Germany. The aim of this study was to investigate the use of CAM and other health promoting substances (e.g., herbal teas) by older adults of at least 70 years of age.

**Methods:**

A cross-sectional questionnaire study was conducted among persons of ≥70 years from metropolitan Berlin and rural parts of Brandenburg, Germany. Recorded were: demographics, current use of CAM, medical diagnoses, users’ opinions and preferences.

**Results:**

A total of 400 older adults, living as ‘self-reliant’ (n = 154), ‘home care service user’ (n = 97), or ‘in nursing home’ (n = 149), and with the legal status ‘without guardian’ (n = 355) or ‘with guardian’ (n = 45) were included (mean age 81.8 ± 7.4 years, 78.5% female). Any type of CAM used 61.3% of respondents (dietary supplements 35.5%, herbal medicines 33.3%, and external preparations 26.8%); 3.0% used drug-interaction causing preparations. Usage was based on recommendations (total 30.3%; in 20.0% by friends or family and 10.4% by pharmacists), own initiative (27.3%), and doctors’ prescription (25.8%). Participants with legal guardian took almost solely prescribed dietary supplements. Of the others, only half (58.7%) informed their general practitioner (GP) of their CAM use. Participants expected significant (44.9%) or moderate (37.1%) improvement; half of them perceived a good effect (58.7%) and two-thirds (64.9%) generally preferred a combination of CAM and conventional medicine. More than half (57.9%) stated that they could neither assess whether their CAM preparations have side effects, nor assess what the side effects might be. Strongest predictors for CAM use were two treatment preferences (vs. ‘conventional only’: ‘CAM only’, OR = 3.98, p = 0.0042 and ‘CAM + conventional’, 3.02, 0.0028) and the type of health insurance (‘statutory’ vs. ‘private’, 3.57, 0.0356); against CAM use two subjective assessments predicted (vs. ‘CAM causes no harm’: ‘CAM causes harmful drug interactions’, 0.25, 0.0536 and ‘I cannot assess side effects’, 0.28, 0.0010).

**Conclusion:**

Older German adults frequently use CAM. They perceived it as an effective complement to conventional medicine, but are not sufficiently informed about risks and benefits.

## Background

Germany has a very long tradition of complementary and alternative medicine (CAM). Many older adults have lifelong experience with herbal medicine and other home remedies due to unavailable conventional care during their childhood. CAM therapies are often used as self-care to enhance wellbeing, to prevent and to cure illnesses [1]. However, the use of CAM by older adults in Germany has not been investigated extensively. In particular, data from older adults under legal guardianship or requiring nursing care are missing, largely, because these groups are hard to reach by conventional survey techniques such as questionnaires and telephone surveys, and health status may preclude responding. Previous studies exploring CAM use in Germany only investigated clients of one private health insurance company [2], excluded person of 70 [3] or 80 [4] years and older, or have not been evaluated specifically for the older adults [5], despite a high, and rising, rate of CAM users among this group (respondents of at least 60 years: 61% in 1970, 73% in 2010) [6,7].

Polypharmacy is also a problem in the geriatric care in Germany and poses a risk for side effects and drug interactions. While seniors at the age of 60 years take 2–3 prescribed medications daily, the number increases among those over 80 years to more than 4–5 drugs per day [8]. Self acquired additional drugs such as herbal medicines or vitamins are not recorded in the statistics of the statutory health insurance because they are not covered. Many herbal drugs and products interactions and side effects are well known, e.g. (i.e., Ginkgo biloba, Valeriana officinalis, St. John’s wort, and grapefruit juice [9,10].

Therefore we investigated the use of CAM and other health promoting substances (e.g., herbal teas) by older adults of at least 70 years, taking care to include under-researched areas such as rural areas or nursing homes.

Our survey solicited information regarding which form(s) of CAM is used and how its use is subjectively assessed, as well as medical context information. We asked for all natural products, drugs and therapies that were taken for treatment or prevention of diseases, this included not only drugs but also medically applied herbal teas and juices. To get the best possible representation of real-life conditions, we included older adults living with a variety of needs for care, living in either a metropolitan or a rural area, and with or without a legal guardian.

## Methods

We conducted a cross-sectional questionnaire study from November 2010 through July 2012. Participation was anonymous and voluntary; participants expressed their agreement through completion of the questionnaire. For those under legal guardianship, the guardian provided legal consent. The study protocol was approved by the ethics committee of the Charité Universitätsmedizin Berlin (EA1/243/09, 2009-11-25 and 2010-12-16).

**Figure 1:**
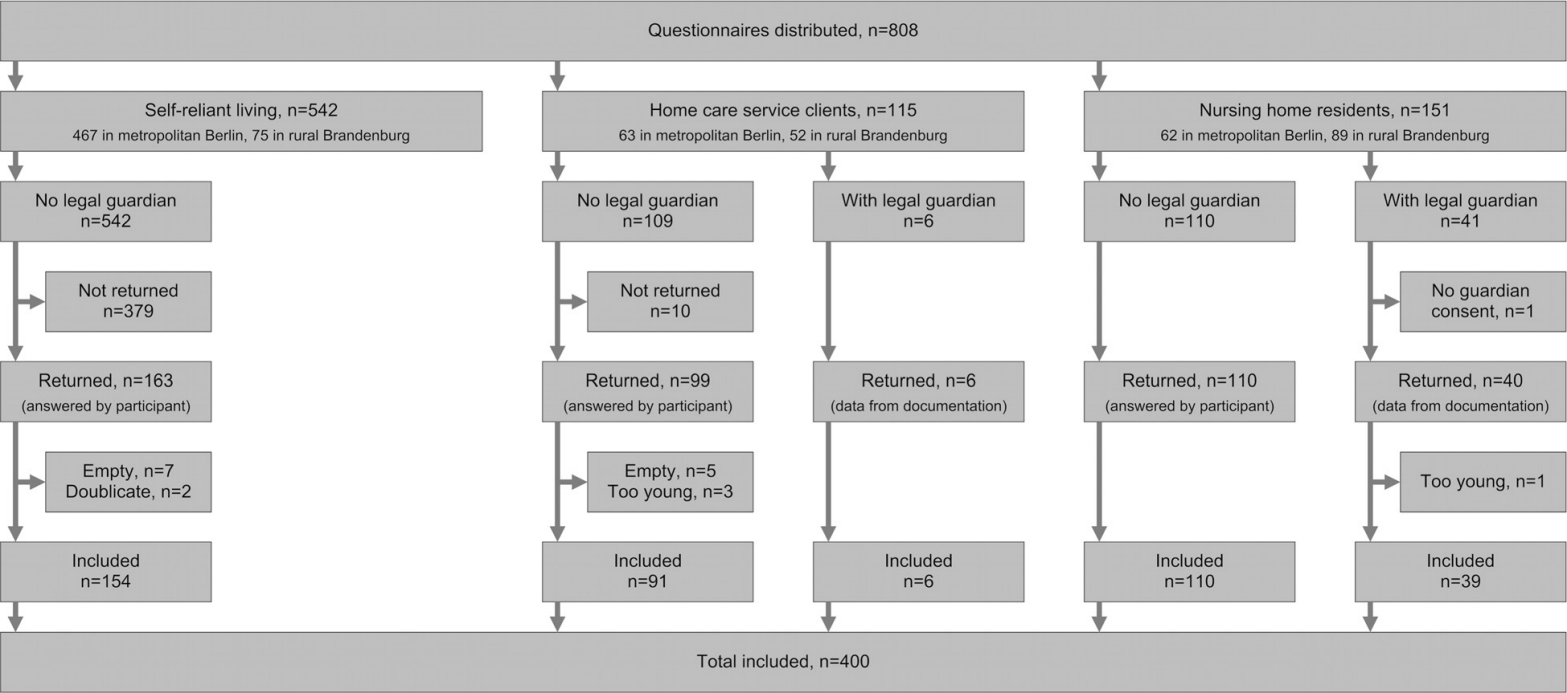
Recruitment, study arms, and inclusion.

Older adults at least 70 years of age living in the states of Berlin (entire city) and Brandenburg (rural northeast, i.e., districts around Berlin including Oberhavel, Barnim, Uckermark and Märkisch-Oderland) were approached through care service providers, nursing homes, community clubs of older adults, or directly through the distribution of questionnaires in mailboxes of senior residential facilities (Berlin only). The care service providers and nursing homes were selected from the phone book and contacted in alphabetical order. The older adults lived in their own homes, either self-reliant or assisted by a home care service, or in a retirement or nursing home. Both home care service users and nursing home residents included older adults with, as well as without, a legal guardian, resulting in 5 study arms (Figure 1). Care service providers selected the clients to be approached by randomized weekday of service and nurse or caregiver, and nursing homes by randomized room numbers. Participants who were able to understand the questionnaires and who were legally permitted to be directly approached answered the questionnaires themselves, with assistance if necessary, and were rewarded with a medical self-care book. For the others (i.e., with legal guardians and not living independently), the caregivers extracted the data from their documentation. Here, no subjective assessments of the participants were asked; service providers or nursing homes received € 4 for every completed questionnaire.

The questionnaire asked for social and demographic data. In this study we also included non-medical health promoting substances such as vitamins or teas under CAM. We recorded for CAM preparations: Name and dosage, reason for the application, on what basis the decision for their use was made, and information regarding participants’ general practitioners (GPs’) knowledge about their patients’ CAM usage. Further items covered the use of non-pharmacological treatments, and, for participants without legal guardians, their subjective opinions and assessments about CAM: expectations towards, experience with, and perceived effects and risks, as well as the generally preferred treatment.

Due to a lack of data about the prevalence of the use of alternative medical drugs in the studied population we assumed a prevalence of 50% of the use of CAM drugs in in the studied population. With an accuracy of 10% for a two-sided confidence interval of prevalence at the 95% level, 96 participants would be needed. In order to achieve this precision, 96 questionnaires would be needed for each of the four groups of older adults: living self reliantly, receiving homecare, living in nursing homes or having a legal guardian. We anticipated that 60% of the self reliant living older adults would return the questionnaires, in all others a return rate of 80% of the questionnaires. Thus the number of self reliant older adults to be contacted was n = 160, for all other groups n = 120. The total number of issued questionnaires thus was n = 520.

The answers from the questionnaires were entered into an electronic database (MS Access™) and checked for plausibility and quality. Descriptive analyses were calculated, and patient groups, stratified by CAM use (yes, no) were compared using chi-square (categorical variables) and t-test (continuous data). For participants without legal guardians, the effect of variables on CAM use was estimated with a multiple logistic regression. The variables were selected on the basis of the calculated p-values and frequencies, as well as an expert’s opinion. Several potential predictor models were tested before the final logistic model was calculated. SPSS 19.0 and higher (© SPSS/IBM) and Statistical Analysis Systems 9.3 (© SAS Institute) were used for randomization and all analyses.

## Results

We contacted 33 care service providers (Berlin 21, Brandenburg 12) and 19 nursing homes (Berlin 7, Brandenburg 12), of which 6 care service providers (Berlin 3, Brandenburg 3) and 6 nursing homes (Berlin 2, Brandenburg 4) participated. Of the 761 questionnaires that were issued to older adults without legal guardian or their nurses, and the 47 questionnaires issued to nurses of older adults with legal guardian, a total of 418 (51.7%) were returned; 400 were entered into the final analysis (Figure 1). The demographic details of the included respondents are presented in Table 1. An equal proportion of included participants lived in rural and metropolitan areas, most were insured by the statutory health insurance, half were approved a care level of the German care insurance system, and three-quarters were female. From the groups without legal guardians, a higher education was more frequently reported in the self-reliant group (Table 1).

**Table 1:** Demographic data

**Demographics**	**Total**	**Self-reliant**	**Home care service**			**Nursing home**		
			**Total**	**Without legal guardian**	**With legal guardian**	**Total**	**Without legal guardian**	**With legal guardian**
	**% (n)**	**% (n)**	**% (n)**	**% (n)**	**% (n)**	**% (n)**	**% (n)**	**% (n)**
Participants^a^	100.0 (400)	38.5 (154)	24.3 (97)	22.8 (91)	1.5 (6)	37.3 (149)	27.5 (110)	9.8 (39)
Age (years, mean ± SD)	81.8 ± 7.4	79.8 ± 7.1	82.1 ± 6.6	82.5 ± 6.1	76.0 ± 10.5	83.8 ± 7.7	83.7 ± 8.2	84.2 ± 6.3
Female	78.5 (310)	78.8 (119)	84.2 (80)	88.8 (79)	28.2 (11)	74.5 (111)	75.5 (83)	71.8 (28)
Living with partner	21.7 (76)	25.3 (38)	24.4 (22)	24.4 (22)	n/a	14.6 (16)	14.6 (16)	n/a
Approved care, any level^c^	58.4 (227)	9.7 (14)	70.5 (67)	70.8 (63)	66.7 (4)	98.0 (146)	98.2 (108)	97.4 (38)
Approved care level I^b,d^	52.4 (118)	85.7 (12)	58.2 (39)	58.7 (37)	50.0 (2)	46.5 (67)	55.7 (59)	21.1 (8)
Approved care level II^b,e^	39.1 (88)	14.3 (2)	38.8 (26)	38.1 (24)	50.0 (2)	41.7 (60)	44.3 (47)	34.2 (13)
Approved care level III^b,f^	8.4 (19)	0 (0)	3.0 (2)	3.2 (2)	0 (0)	11.8 (17)	0 (0)	44.7 (17)
Statutory health insurance	93.1 (359)	85.7 (126)	96.7 (87)	96.5 (82)	100.0 (5)	98.7 (146)	99.1 (109)	97.4 (37)
Private health insurance	6.8 (26)	15.3 (21)	3.3 (3)	3.5 (3)	0 (0)	1.4 (2)	0.9 (1)	2.6 (1)
>10 years of school	12.1 (42)	21.1 (31)	5.6 (5)	5.6 (5)	n/a	5.5 (6)	5.5 (6)	n/a
Metropolitan area (Berlin)	50.3 (201)	61.7 (95)	47.4 (46)	44.0 (40)	100.0 (6)	40.3 (60)	47.3 (52)	20.5 (8)
Rural area (Brandenburg)	49.7 (199)	38.3 (59)	52.6 (51)	56.0 (51)	0 (0)	59.7 (89)	52.7 (58)	79.5 (31)

Percent of valid answers of the respective group; ^a^percent of total study population; ^b^percent of older adults with approved care level.

^c^Care levels according to German law (§ 15 SGB XI). ^d^Requires help ≥1×/d for ≥2 performances with body care, food or mobility, plus several times per week with household. The average duration of ≥90 min/d includes ≥45 min of basic care [11]. ^e^Requires help ≥3×/d at different times with body care, food or mobility, plus several times per week with household. The average duration of ≥3 hrs/d includes ≥2 hrs of basic care [12]. ^f^Requires help around the clock with body care, food or mobility, plus several times per week with household. The average duration of ≥5 hrs/d includes ≥4 hrs of basic care [13].

Nearly two out of three participants (61.3%) used at least one CAM preparation (Table 2). Both highest and lowest rates were seen in home care service clients (78.0% without, and 33.3% (2 of 6) with legal guardians). Most frequently they took dietary supplements (35.5%) and herbal medicines (33.3%). Physical therapy (41.3%) led the non-pharmaceutical therapies. Cardiovascular diseases were the most frequent reason for medication of any kind (26.8%), followed by chronic pain (24.5%). Table 2 shows details for all subgroups.

**Table 2:** Use of CAM preparations, non-pharmaceutical therapies, and underlying diseases

**Use**	**Total**	**Self-reliant**	**Home care service**			**Nursing home**		
			**Total**	**Without legal guardian**	**With legal guardian**	**Total**	**Without legal guardian**	**With legal guardian**
	**% (n)**	**% (n)**	**% (n)**	**% (n)**	**% (n)**	**% (n)**	**% (n)**	**% (n)**
Use of CAM preparations								
Any preparation	61.3 (245)	52.6 (81)	75.3 (73)	78.0 (71)	33.3 (2)	61.1 (91)	57.3 (63)	71.8 (28)
Dietary supplements	35.5 (142)	31.2 (48)	45.4 (44)	48.4 (44)	0 (0)	33.6 (50)	22.7 (25)	64.1 (25)
Herbal therapy	33.3 (133)	35.7 (55)	52.6 (51)	56.0 (51)	0 (0)	18.1 (27)	23.6 (26)	2.6 (1)
External applications	26.8 (107)	16.9 (26)	41.2 (40)	41.8 (38)	33.3 (2)	27.5 (41)	34.5 (38)	7.7 (3)
Homeopathy	8.0 (32)	11.7 (18)	10.3 (10)	11.0 (10)	0 (0)	2.7 (4)	3.6 (4)	0 (0)
Juices (vegetable/fruit)	1.5 (6)	1.3 (2)	3.1 (3)	3.3 (3)	0 (0)	0.7 (1)	0.9 (1)	0 (0)
Other	1.5 (6)	1.9 (3)	0 (0)	0 (0)	0 (0)	2.0 (3)	1.8 (2)	2.6 (1)
**Diseases for which medication is used**								
Cardiovascular diseases	26.8 (107)	31.8 (49)	40.2 (39)	42.9 (39)	0 (0)	12.8 (19)	17.3 (19)	0 (0)
Chronic pain	24.5 (98)	27.9 (43)	38.1 (37)	39.6 (36)	16.7 (1)	12.1 (18)	15.5 (17)	2.6 (1)
Gastrointestinal diseases	14.0 (56)	16.2 (25)	24.7 (24)	26.4 (24)	0 (0)	4.7 (7)	6.4 (7)	0 (0)
Endocrine diseases	10.3 (41)	10.4 (16)	18.6 (18)	18.7 (17)	16.7 (1)	4.7 (7)	6.4 (7)	0 (0)
Psychological disorders	7.0 (28)	9.1 (14)	12.4 (12)	13.2 (12)	0 (0)	1.3 (2)	1.8 (2)	0 (0)
Metabolic diseases	3.3 (13)	5.8 (9)	3.1 (3)	3.3 (3)	0 (0)	0.7 (1)	0.9 (1)	0 (0)
Other	45.3 (181)	42.9 (66)	57.7 (56)	60.4 (55)	16.7 (1)	39.6 (59)	47.3 (52)	17.9 (7)
Not stated	5.0 (20)	n/a	0 (0)	n/a	0 (0)	13.4 (20)	n/a	51.3 (20)
**Use of non-pharmaceutical therapies**								
Any therapy	60.5 (242)	50.7 (78)	64.9 (63)	65.9 (60)	50.0 (3)	67.8 (101)	69.1 (76)	64.1 (25)
Physical therapy	41.3 (165)	50.6 (78)	49.5 (48)	49.5 (45)	50.0 (3)	20.1 (39)	23.6 (26)	33.3 (13)
Acupuncture/Chinese Medicine	4.5 (18)	9.1 (14)	4.1 (4)	4.4 (4)	0 (0)	0 (0)	0 (0)	0 (0)
Chiropractic/manual therapy/osteopathy/physiotherapy	4.5 (18)	7.8 (12)	6.2 (6)	6.6 (6)	0 (0)	0 (0)	0 (0)	0 (0)
Occupational therapy/logopedics	2.8 (11)	n/a	1.0 (1)	n/a	16.7 (1)	6.7 (10)	n/a	25.6 (10)
Other	35.8 (143)	20.8 (32)	34.0 (33)	35.2 (32)	16.7 (1)	52.3 (78)	57.3 (63)	38.5 (15)

Percent of valid answers of the respective group. Multiple answers allowed.

The CAM preparations most frequently used by older adults without legal guardians are listed in Table 3. Some of them are known to cause drug interactions (i.e., Ginkgo biloba, Valeriana officinalis, St. John’s wort, and grapefruit juice [9,10]); such preparations were used by 3.0% of the participants (Table 3).

**Table 3:** CAM preparations used most frequently by older adults without legal guardians

**CAM preparation**	**% (n)**
**Homeopathy**	
Schuessler Salts	3.1 (11)
**Dietary supplements**	
Minerals	
Magnesium	13.5 (48)
Calcium	9.0 (32)
Zinc	2.5 (9)
Iron	2.0 (7)
Single vitamins	
Vitamin C	2.3 (8)
Vitamin B	1.4 (5)
Vitamin D	0.8 (3)
Combination (mineral/vitamin/other)	4.5 (16)
**Herbs**	
Without specific indication	
Chamomile (Matricaria recutita) tea	6.5 (23)
Unspecified “herbal” tea	5.1 (18)
Fennel, anise, caraway (Foeniculum vulgare, Pimpinella anisum, Carum carvi) tea	4.8 (17)
Peppermint (Mentha piperita) tea	3.7 (13)
Medicinal formulation	
Bronchial tea	2.8 (10)
Kidney-Bladder tea	2.5 (9)
Gastrointestinal tea	2.3 (8)
Ginkgo (Ginkgo biloba)	3.9 (14)
Valerian (Valeriana officinalis)	2.8 (10)
St. John’s wort (Hypericum perforatum)	1.4 (5)
**External applications**	
Ointments	
Arnica (Arnica montana)	5.6 (20)
Calendula (Calendula officinalis)	3.1 (11)
Mountain pine (Pinus Montana) foot cream/balm/footbath	3.1 (11)
Rubbing alcohol	3.1 (11)
**Juices**	
Unspecified “vegetable” juice	0.6 (2)
Grapefruit (Citrus paradisi) juice	0.3 (1)

CAM (as defined in this study, see text) preparations that were most frequently used in the respective category. Percent of 355 older adults without legal guardian. Multiple answers possible; 751 answers in total.

Of the older adults with legal guardians, 88.9% used CAM. In 92.9% of cases, their GPs prescribed the preparations but only 51.3% documented the reason. Users took predominantly dietary supplements: Vitamin D_3_ (22.2%), vitamin B_12_ (20.0%), folic acid (13.3%), calcium (6.7%), magnesium (2.2%), and iron (2.2%) and only in 2.2% the herbal preparation, valerian. CAM users without legal guardians expected a marked (44.9%) or moderate (37.1%) improvement of their conditions; 11.7% did not state their expectations. More than half (58.7%) of the users experienced a good effect, 27.4% only a minor effect, and 6.0%, no effect.

CAM usage was in 31.3% based on recommendations (10.4% by pharmacists, 20.0% by friends or family) or as a result of one’s own initiative (27.3%). Only a quarter (25.8%) of the total CAM uses were prescribed by medical or nonmedical practitioners (in Germany ‘Heilpraktiker’) (Table 4). More than half (58.7%) of CAM users informed their GPs of their CAM uses. Merely 16.6% of all participants were asked about their CAM usage by their GPs – the more dependent their living situation, the less frequently their GPs inquired (Table 4). Of the older adults without legal guardians, more than half (57.9%) stated that they could neither assess whether or not their CAM preparations would have side effects, nor what side effects these might be, and only 5.0% were aware of possibly harmful drug interactions (Table 4). Nearly two-thirds (64.9%) of this group preferred a combination of CAM and conventional medicine (Table 4).

**Table 4:** Considerations, decision, and information about CAM use

	**Total**	**Self-reliant**	**Home care service users**			**Nursing home residents**		
			**Total**	**Without legal guardian**	**With legal guardian**	**Total**	**Without legal guardian**	**With legal guardian**
	**% (n)**	**% (n)**	**% (n)**	**% (n)**	**% (n)**	**% (n)**	**% (n)**	**% (n)**
**Participants’ assessment of side effects of CAM preparations**								
Cannot assess	57.9 (187)	54.2 (71)	48.2 (40)	48.2 (40)	n/a	69.7 (76)	69.7 (76)	n/a
No harm	23.5 (76)	15.3 (20)	36.1 (30)	36.1 (30)	n/a	23.9 (26)	23.9 (26)	n/a
Mild side effects	13.6 (44)	24.4 (32)	10.8 (9)	10.8 (9)	n/a	2.8 (3)	2.8 (3)	n/a
Harmful in combination with other drugs	5.0 (16)	6.1 (8)	4.8 (4)	4.8 (4)	n/a	3.7 (4)	3.7 (4)	n/a
**General treatment preference**								
CAM and conventional medicine	64.9 (209)	73.9 (96)	59.3 (51)	59.3 (51)	n/a	58.5 (62)	58.5 (62)	n/a
Only conventional medicine	18.9 (61)	19.2 (25)	15.1 (13)	15.1 (13)	n/a	21.7 (23)	21.7 (23)	n/a
Only CAM	16.2 (52)	6.9 (9)	25.6 (22)	25.6 (22)	n/a	19.8 (21)	19.8 (21)	n/a
**CAM preparation use is based on**								
Recommendation - total	31.3 (125)	30.5 (47)	43.3 (42)	46.2 (42)	0 (0)	24.2 (36)	32.7 (36)	0 (0)
- by pharmacist	10.4 (36)	9.3 (14)	n/a	12.4 (36)	n/a	n/a	10.4 (11)	n/a
- by friends and family	20.0 (69)	14.7 (22)	n/a	25.8 (23)	n/a	n/a	22.6 (24)	n/a
Own initiative	27.3 (109)	26.6 (41)	44.3 (43)	45.1 (41)	33.3 (2)	16.8 (25)	22.7 (25)	0 (0)
Prescription	25.8 (103)	20.1 (31)	25.8 (25)	27.5 (25)	0 (0)	47 (31.5)	19.1 (21)	66.7 (26)
**GP information about CAM preparation use**								
Participant informed GP^a^	58.7 (138)	62.3 (48)	49.3 (34)	48.5 (33)	50.0 (1)	62.9 (56)	45.9 (28)	100.0 (28)
GP inquired about use	16.6 (53)	21.1 (27)	19.5 (16)	19.5 (16)	n/a^b^	9.2 (10)	9.2 (10)	n/a^b^

Percent of valid answers of respondents, ^a^Percent of 245 users. ^b^Prescription mandatory for all medicines including CAM. For definition of ‘CAM’ in this study see text.

**Figure 2:**
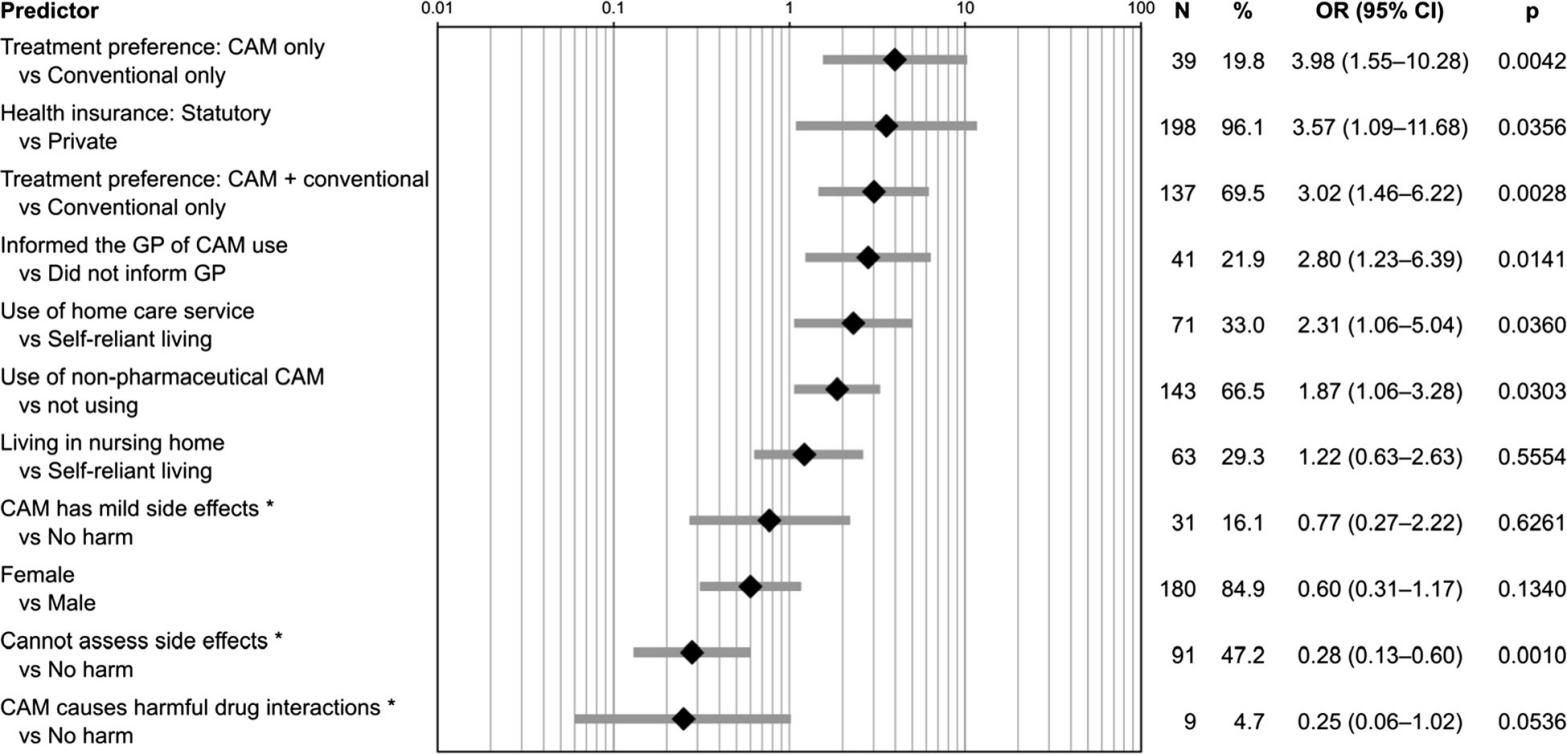
**Predictors for CAM use.** Multiple logistic regression estimating the effect of variables on CAM (as defined for this study, see text) use. Results for participants without a legal guardian, * = participants’ assessment. Predictor model selected on the basis of p-values and frequencies as well as an expert’s opinion. Log scale; OR = odds ratio; CI = confidence interval.

The variables that predict the use of CAM preparations for participants without legal guardians are shown in Figure 2. Highest odds ratios predicting CAM use were found for two treatment preferences (CAM only, OR = 3.98, p = 0.0042; CAM + conventional, 3.02, 0.0028) and the type of health insurance (statutory, 3.57, 0.0356). For those against CAM use, two subjective assessments were the strongest predictors (CAM causes harmful drug interactions, 0.25, 0.0536; I cannot assess side effects, 0.28, 0.0010). Gender (female, 0.6, 0.1340) and the degree of independence of the living situation (in nursing home, 1.22, 0.5554; using home care service, 2.31, 0.0360) were found to be weaker predictors.

## Discussion

In an anonymous questionnaire survey in Germany of adults aged 70 years and older, we generally found a very high rate of CAM users. The older adults regarded CAM as an effective therapeutic approach with low risks of side effects. Predominantly dietary supplements and herbal preparations were applied, mostly without a physician’s prescription.

One strength of this study is the coverage of naturalistic settings: metropolitan and rural areas in both former ‘East’ and ‘West’ Germany, a broad range of the need for care, and participants with and without legal guardians. Our recruitment strategy facilitated the inclusion of multi-morbid older adults and thus reduced the selection bias for healthy respondents, which made it preferable to a phone survey or identifying possible participants through records from registration offices. The practice of directly approaching participants resulted in a high return rate in participants without legal guardianship, as has been suggested elsewhere [14].

The results obtained were collected in Berlin and Brandenburg (Germany). It is unclear whether they can be transferred to other German states. The recruitment of older adults with legal guardians was less successful than expected. The additional work for their nurses (obtaining guardian consent, data extraction) and possibly other concerns established an entry barrier that resulted in less than the 100 required participants; thus not all planned statistics could be calculated.

In all groups it is possible that some older adults listed all of their diseases as reasons for CAM use. Also, results from nursing homes may have been influenced by the fact that some of the homes offered CAM treatments, which may be interpreted as a distortion of the results or as part of the environment. A few respondents who were much younger than the intended inclusion age made it necessary to relax the strict intention-to-treat approach and exclude these untargeted extreme outliers.

A comparison of our results with the existing research is difficult because of the small body of literature and its different eligibility criteria. For Germany, only one anonymous questionnaire survey by Büssing et al. exists [2], which included only privately insured elderly without a legal guardian. Participants in that study were, on average, 17 years younger (mean age 64.7 ± 11.2 years) than our participants. These facts contribute to the other marked demographic differences between that and the present study: females were 30% of their respondents vs. 79% of ours, 53% (vs. 12%) had attended school for >10 years, 82% (vs. 22%) lived with a partner. More than two-thirds (68%) were healthy. The survey asked for prescribed drugs only. Private health insurance in Germany can cover all CAM expenditures, whereas statutory insurance does not, and a large number of our respondents were covered by statutory insurance. Therefore the differences in CAM use in the Büssing et al. study [2] to our results ought to be interpreted with great caution: More of the older adults they surveyed had used acupuncture/Chinese medicine (21% vs. 5%), homeopathy (21% vs. 8%), osteopathy/physiotherapy (12% and 19% vs. 5%), and fewer used phytotherapy (7% vs. 33%).

Another German survey of the general population included persons aged 18 to 79 years [4]. It allows a rough comparison of this age group with the CAM use of the general population, although the different survey methods prohibit detailed conclusions. We found a lower percentage of participants using homeopathy (8% vs. 17% in the last 12 months), acupuncture/Chinese medicine (4.5% vs. 6% in the last 12 months), or chiropractic/osteopathy (4.5% vs. 6% in the last 12 months). The motivation to use of natural/herbal medicines and homeopathy was much lower than in the general population. On their own initiative, 27% used any CAM, as opposed to 55% (CAM preparations excluding homeopathy) and 47% (homeopathy) in our study.

A general German population survey by the Allensbach Institute [7] also found that the risk of side effects of CAM was regarded as low.

Worldwide, only 5 studies on CAM use in residential care settings appear to have been published [15]. Their results are not comparable to our study because they include either a very small population (n = 6, assisted living, Australia [16]), or are restricted to specific ailments or treatments (dementia, Australia [17]; pain, UK [18]; T’ai chi, Taiwan [19]; TCM, Hong Kong [20]). For the other settings investigated in our study, we were able to identify 5 studies that were all conducted in the USA, where spiritual practices, mind-body techniques, and the use of megavitamins (uncommon in Germany) are subsumed under CAM, which limits comparability.

A cross-sectional survey by Cherniack et al. [14] with 421 interviewees found a 12 month CAM use prevalence of 58%. Female gender, higher education, and thyroid disease or arthritis correlated with CAM use. The cross-sectional questionnaire survey by Cheung et al. [21] among 1200 randomly selected metropolitan adults aged ≥65 years recorded 63% CAM users. In our study, use was lower for nutritional supplements (36% vs. 44% and 28% megavitamins) and chiropractic (5% vs. 18%), but higher for herbal medicine (33% vs. 21%). Although 80% of the Cheung et al. participants were satisfied with CAM, only 53% (vs. 59%) informed their physicians of the use.

Another cross-sectional analysis by Cohen et al. [22] from a geriatrics outpatient department found that 64% of the participants reported the use of dietary supplements or herbs, but use was documented for only 35%. Another Australian study investigated the use of complementary and alternative medicines in a group of older rural Australian attending a multi-disciplinary health screening clinic. Three-quarters (78%) of respondents had used at least one CAM product within the past 12 months and 66% had visited a CAM practitioner. Almost half (46%) had not discussed their use of CAM with their doctor and only 15% had discussed their CAM use with a pharmacist [23]. The Ginkgo Evaluation of Memory (GEM) study [24] also recorded the use of CAM drugs and dietary supplements, but its exclusion criteria prohibit detailed comparisons. Its participants predominantly used dietary supplements, whereas our population used these and herbal preparations (mostly teas) in equal proportion.

A randomized subsample (n = 1099) of the 2000 wave of the Health and Retirement Study [25] answered questions about their CAM use. The evaluation included subgroups in the age ranges of 65–79 (43%) and 80 years or older (14%). The use of CAM was more frequent than in our study (87% of the first group, 92% of the latter, vs. 61%), but included lifetime prevalence for chiropractic and alternative practitioner consultations. Non-herbal dietary supplements were much more frequently used than in our study (60%, 70% vs. 36%), most often multivitamins (48%, 51%, not seen in our study), vitamin A (12%, 9%, ditto), vitamin C (30%, 35% vs. 2%), vitamin D (15%, 13% vs. 1%), vitamin E (35%, 39%, not seen in our study), calcium (31%, 38% vs. 9%). Magnesium was an exception (13%, 12% vs. 14%). Herbal therapies (21%, 8%) and supplements (20%, 18%) were less commonly used (33% of our participants). Higher age correlated with the use of dietary supplements, higher education with the use of dietary and herbal supplements.

In our study we found none of the predictors for, or correlations with, the use of CAM preparations or therapies that had been seen in some of the more or less differing populations cited above [25], but we observed a general similarity that raises concerns about drug safety. More than half of our study participants stated that they could neither assess whether their CAM preparations would have side effects, nor assess what side effects might arise. Only 5.0% were aware of possibly harmful drug interactions. In many of those cases the primary care physicians were also insufficiently informed about the use of CAM preparations [21]. If they are not aware of this “substantial concomitant use of prescription drugs and dietary supplements” [24], harmful interactions of drugs with herbs or supplements cannot be prevented. Conversely, physicians who prescribe CAM treatments may meet the needs of older adults. This can be seen as a typical problem for health systems where CAM medications or CAM therapies are not included in the statutory health insurance system and thus their use cannot be sufficiently monitored. One way to increase the safety of CAM drug use in Germany would be to reimburse the expenses for CAM medication by the statutory health insurance system, as it was common in Germany until 2002. GPs would again be able to inform patients and also control CAM medication at least to a certain degree, which could help to minimize the risk of potential side effects or drug interactions.

## Conclusion

In this first study that included participants in living situations involving various degrees of independence we found a high rate of users of CAM preparations and dietary supplements among German older adults. Self-reliant older adults primarily use dietary supplements, herbal medicines and external preparations. For the most part they follow recommendations by pharmacists, friends or relatives or make their own decisions, whereas older adults with legal guardians or a high need for care take prescribed vitamins and minerals. General practitioners were insufficiently informed about CAM usage. Older adults perceived CAM as an effective complement to conventional medicine, but were not sufficiently informed about risks.

## Competing interests

The authors declare that there are no conflicting interests regarding the publication of this article. This study was funded by the Karl and Veronica Carstens Foundation (Essen, Germany).

## Authors’ contributions

KS participated in study concept and design, data collection, statistical analysis, data interpretation, and manuscript drafting. SB did the statistical analysis and participated in data interpretation and manuscript drafting. MT participated in study concept and design, data interpretation, and manuscript drafting. CMW participated in study concept and design, data interpretation, and manuscript drafting. All authors read and approved the final manuscript.

## Pre-publication history

The pre-publication history for this paper can be accessed here:

http://www.biomedcentral.com/1471-2318/14/38/prepub
